# The Trickle-Down Effect of Leaders’ Pro-social Rule Breaking: Joint Moderating Role of Empowering Leadership and Courage

**DOI:** 10.3389/fpsyg.2018.02647

**Published:** 2019-01-07

**Authors:** Yushuai Chen, Lan Wang, Xin Liu, Hong Chen, Yunyang Hu, Hongling Yang

**Affiliations:** ^1^Department of Applied Psychology, Guangdong University of Foreign Studies, Guangzhou, China; ^2^School of Management, Jinan University, Guangzhou, China; ^3^Sun Yat-sen Business School, Sun Yat-sen University, Guangzhou, China; ^4^School of Management, Guangdong Industry Polytechnic, Guangzhou, China

**Keywords:** pro-social rule breaking, empowering leadership, courage, social learning theory, humanistic cognitive behaviorism theory

## Abstract

Based on social learning theory and humanistic cognitive behaviorism theory, this study examined the trickle-down effect of leader PSRB and its boundary conditions. We proposed a three-way interaction of leader PSRB, empowering leadership, and follower courage to predict follower PSRB. Data were collected from 174 leader-follower dyads. Multiple moderated regressions (MMR) revealed that leader PSRB was positively related to follower PSRB, and that the effect was stronger under conditions of high empowering leadership or high courage. A three-way interaction effect suggested that the positive relationship between leader PSRB and follower PSRB was strongest when both empowering leadership and courage were high. Finally, the theoretical and practical implications were discussed.

## Introduction

The concept of “rule-breaking” in the organizational literature is defined as deviant behavior detrimental to an organization and its members (Bennett and Robinson, 2000). However, as positive psychology has emerged, scholars have begun focusing on pro-social motivations behind the rule violations and have offered the concept of “Pro-Social Rule Breaking” (PSRB). PSRB refers to behaviors intended to violate a formal organizational policy/regulation to promote the welfare of the organization or one of its stakeholders (Morrison, 2006). Empirical studies have explored the antecedents of PSRB, including individual factors, such as empathy, proactive personality, risk-taking propensity and conscientiousness (Morrison, 2006; Dahling et al., 2012), and situational factors, such as job demands, co-worker PSRB, and transformational leadership (Dahling et al., 2012; Huang et al., 2014). However, we believe that added insight can be provided by investigating leaders’ behaviors as additional antecedents.

Social information processing model (Salancik and Pfeffer, 1978) posits that individuals make decisions and exhibit subsequent behaviors according to the information or clues that they obtain from their surroundings. Leaders, as important clues of organizational environment, are crucial influences on follower behaviors (Bavik et al., 2018). Previous studies have examined ways that leadership style influenced follower PSRB (Huang et al., 2014; Zhu et al., 2018), while ignoring the trickle-down effect of leader PSRB on follower PSRB. According to social learning theory (Bandura, 1977), leaders are powerful and hold high-level authoritative positions in organizations, which make them easily become role models, and their behaviors are easily learned and imitated by followers. In this way, leaders’ behaviors might have trickle-down effects that induce similar responses in their followers (Wang et al., 2017). Following this logic, it is important to consider whether followers regard leader PSRB as a model behavior to further motivate their own PSRB. This is the first issue we aim to explore.

However, trickle-down effects might not always exist, and they might depend on observers’ characteristics and contextual factors (Bandura, 1977). Therefore, we infer that there have boundary conditions on the relationship between leader PSRB and follower PSRB. To date, research have found that empowering leadership improved followers’ self-efficacy and create a supportive and autonomous working environment (Arnold et al., 2000; Zhang and Bartol, 2010), which may increase the likelihood that they will deviate from organizational rules in some instances. Thus, we predict that followers are more likely to learn and enact PSRB when empowering leadership is high. In other words, empowering leadership might moderate the relationship between leader PSRB and follower PSRB.

In addition, the consequences of PSRB might be uncertain (Morrison, 2006). In the face of uncertainty and unknown situations, people usually must choose either to confront or to avoid, courage drives people to confront uncertain and unknown situations and believe that their actions can lead to positive outcomes (Woodard, 2004; Goud, 2005). Hence, we further predict that courage moderates the relationship between leader PSRB and follower PSRB.

**FIGURE 1:**
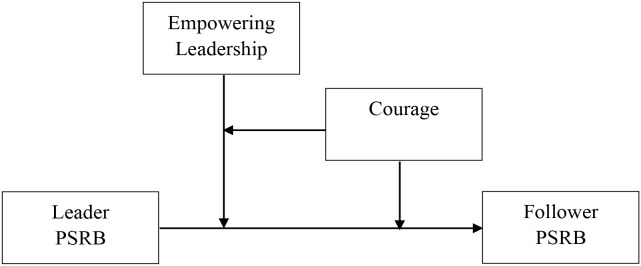
Conceptual model of the study.

In sum, as depicted in Figure 1, the main purpose of this study was to investigate whether leader PSRB can be transmitted to followers as well as identify the boundary conditions on the trickle-down effect. We expected to advance the previous research in two ways.

First, some studies have explored the antecedents of follower PSRB, such as conscientiousness, proactive personality, transformational leadership, and so forth (e.g., Morrison, 2006; Dahling et al., 2012; Huang et al., 2014). However, the effect of leader PSRB on follower PSRB is still unexplored. We attempt to address this gap in the literature by examining the trickle-down effect of leader PSRB.

Second, previous trickle-down research has identified individual characteristics (e.g., Aryee et al., 2007) or contextual factors (e.g., Mawritz et al., 2012; Ambrose et al., 2013) that limit or enhance the trickle-down influence. Yet, few studies have considered the joint effects of individual characteristics and contextual factors on trickle-down processes. Therefore, this study address this question by examining a three-way interaction effect of leader PSRB, empowering leadership (contextual factor) and courage (individual characteristic) on follower PSRB. By doing so, we attempt to offer a new perspective on the boundary conditions in the trickle-down processes.

### Theories and Hypotheses

#### PSRB

Violations of formal organizational rules are generally understood as self-interested, deviant, or unethical workplace behaviors (Vardaman et al., 2014). However, a more nuanced perspective recently has emerged. Morrison (2006) introduced the PSRB construct to explain rule breaking not motivated by deviant intentions, but prompted by a desire to help the organization to meet its objectives instead. Morrison (2006) defined PSRB as “behaviors that intentionally violate a formal organizational policy and regulation for the sake of promoting the welfare of the organization or one of its stakeholders.” For example, consider a hypothetical waiter dealing with an upset customer. A common response might be to offer the customer a free dessert in an attempt to salvage the situation and satisfy the customer. Although offering free food might violate an organizational rule, in this context the rule breaking is in the greater interest of the organization; the customer is now appeased and is likely to remain a repeat customer, and the benefits of this continued business far outweigh the cost of the free item (Dahling et al., 2012).

PSRB has several important characteristics. First, PSRB involves violations of top-down rules and policies set by the organization rather than deviation from the emergent and informal norms of social groups. Second, PSRB is voluntary rule-breaking behavior and individuals have the power to decide whether or not to engage in it. Third, PSRB is deliberate violation of explicit, active rules; violations accidentally committed out of ignorance are not categorized as PSRB behaviors. Finally, the main motivation for PSRB is to help the organization or its stakeholders (Morrison, 2006; Dahling et al., 2012).

#### From Leader PSRB to Follower PSRB

Social learning theory (Bandura, 1977) argues that individuals learn social behaviors by observing behaviors of credible role models. Based on this theory, followers probably learn PSRB by observing other organizational members’ PSRB. Morrison (2006) found that co-worker PSRB functioned as a role model that influenced follower PSRB. However, leaders are more likely than co-workers to be role models because of their relatively higher authority and competence (Wang et al., 2017). Therefore, we posit that leader PSRB might have trickle-down effects on followers through social learning mechanism.

First, the extent of a role model’s attraction to observers plays an important part in the success of the observational learning process. Leaders generally have higher positions than followers have in the organizational hierarchy, and possess more resources and power. In addition, leaders might crucially influence followers’ promotions and pay increases (Dépret and Fiske, 1993). Therefore, leaders might be extraordinarily attractive to followers, which might increase the likelihood that followers pay attention to their work behaviors. Second, when leaders perform PSRB, they are signaling that the PSRB is permissible, and followers subconsciously learn the value criteria behind the PSRB, which is that the organizational rules can be broken on behalf of the welfare of the organization and its stakeholders. Guided thereby, followers are more likely to perform similar PSRB when faced with similar situations. Thus, we predict that followers are more inclined to perform PSRB once observing their leaders’ PSRB.

Hypothesis 1: Leader PSRB positively relates to follower PSRB.

#### The Moderating Role of Empowering Leadership

Empowering leadership is a leadership process in which leaders share powers and enhance followers’ motivation at work by explaining work meaning, encouraging followers to participate in decision-making, showing more confidence in followers’ delivering good performance, and allowing job autonomy (Arnold et al., 2000; Zhang and Bartol, 2010). Spreitzer and Doneson (2005) found that empowering leadership was a crucially important factor driving followers to perform positive deviance, of which PSRB is one type. Therefore, we expected empowering leadership to have an important influence on follower PSRB. First, when the extent of empowering leadership is high, followers are more likely than when it is low to recognize that their jobs are important, which, in turn, increases their willingness to take risks and seek innovation and change. Second, empowering leadership helps to create an unrestricted and low-stress atmosphere where followers feel that they can work in the ways they prefer (Spreitzer and Doneson, 2005), which makes it possible for followers to perform PSRB. Third, when the extent of empowering leadership is high, leaders have strong confidence in their followers’ performances, which helps to increase followers’ sense of self-efficacy (Srivastava et al., 2006) enabling them to handle less favorable situations with positive attitudes. Self-efficacy and positivity are important psychological resources that might develop followers’ positive thinking and expectations regarding the consequences of their behaviors, which might encourage them to perform PSRB (Seligman et al., 2005). Finally, followers tend to appraise highly empowering leadership as relatively more innovative, positive, and encouraging, making those leaders and their behaviors relatively more attractive, thereby increasing followers’ willingness to imitate and learn leader PSRB.

In contrast, when the extent of empowering leadership is low, leaders almost demand that followers focus on obedience, which reduces job autonomy (Spreitzer and Doneson, 2005), and followers’ likelihood to perform PSRB. Furthermore, because it is unlikely that followers would consider a low level of empowering leadership as positive and innovative, they would be unlikely to appreciate or trust their leaders (Gao et al., 2011). Therefore, even if followers observed leader PSRB, in the context of low levels of empowering leadership, they would not be likely to imitate and learn it. Therefore, we put forward the following hypothesis.

Hypothesis 2: Empowering leadership moderates the positive relationship between leader PSRB and follower PSRB, such that the positive relationship is stronger when the extent of empowering leadership is high.

#### The Moderating Role of Courage

Courage is defined as the voluntary willingness to act, with or without varying levels of fear, in response to a threat to achieve an important, perhaps moral, outcome or goal. It is a kind of personal trait which has two important components: threat and worthy or important goal (Woodard and Pury, 2007; Wang, 2011). PSRB has the important goal of protecting the interests of the organization and/or its stakeholders and, simultaneously, it is accompanied by high risk (a threat). Therefore, PSRB comprises behaviors driven by courage, and we expected that followers with relatively more courage would be more likely to perform PSRB after learning leader PSRB through observation.

First, according to the definition of courage, followers with high courage prefer to pursue moral goals, and pro-social behavior can be understood as a concrete embodiment of moral goals (Hannah and Walumbwa, 2011). Therefore, followers with high courage tend to exhibit more pro-social behavior (Hannah and Walumbwa, 2011). Second, when perceiving an apparent threat, courageous followers are less fearful and anxious and more confident when dealing with possible negative outcomes than their meek counterparts (Seligman et al., 2005). In other words, although courageous followers know that PSRB might inflict negative results, such as punishment (Morrison, 2006; Dahling et al., 2012), they still are highly likely to perform PSRB because they have the ability to regulate the fear, lessen their anxiety, and handle any negative or even catastrophic outcomes of their rule-breaking behaviors.

In contrast, timid followers are too fearful and anxious to perform PSRB because they cannot control their fear of a threatening situation or the possible negative results of violating the rules (Seligman et al., 2005), they tend to be obedient and conservative (Woodard and Pury, 2007). Hence, even if leaders demonstrated PSRB, these followers would not imitate them because they would safely choose to comply with the organizational rules rather than break them for the organization’s best interests. Furthermore, followers with low courage have a lower motivation to pursue a moral goal. Therefore, he or she is less likely to exhibit pro-organizational behavior. Based on this reasoning, we hypothesized as follows.

Hypothesis 3: The extent of courage moderates the positive relationship between leader PSRB and follower PSRB, such that the positive relationship is stronger when the extent of courage is high.

#### The Interaction Effects of Empowering Leadership and Courage

Human cognitive behaviorism theory posits that any action taken in response to a risky situation could be influenced by interactions among courage, cognition and environment (Gruber, 2011). As leaders perform PSRB, followers will learn the value criteria embodied in PSRB that the organizational rules can be broken to facilitate the welfare of the organization and its stakeholders. Hence, based on human cognitive behaviorism theory, we hypothesized that courage, value criteria (cognition) formed by observing leader PSRB and empowering leadership (environment) would have interaction effects on follower PSRB. Specifically, of the four possible combinations (high courage and high empowering leadership, high courage and low empowering leadership, low courage and high empowering leadership, and low courage and low empowering leadership), the relationship between leader PSRB and follower PSRB would be strongest when courage and empowering leadership were high for the following reasons.

When the extents of courage and empowering leadership are high, followers are more likely than when they are low to transform the symbolic representation of learned PSRB into actual behaviors because they have firm beliefs, the ability to regulate fear, and an external context (an unrestricted and relaxed atmosphere) that encourage PSRB (Carmeli et al., 2011). When the extent of follower courage is high and the extent of empowering leadership is low, leaders tend to rigidly control their followers, a condition under which even courageous followers would have difficulty confronting threats and taking risks because they would be discouraged from performing PSRB in a work context of low autonomy and freedom. When the extent of follower courage is low and the empowering leadership is high, despite leaders’ tendencies to encourage followers to make decisions on various matters, timid followers would be afraid to take actions that might have any negative results and they would be less likely to engage in PSRB. Finally, when the extents of follower courage and empowering leadership are low, leader PSRB would have the weakest trickle-down effects on follower PSRB because followers would have neither the courage nor the autonomy to enact PSRB. Ultimately, we hypothesized the following interaction effects on the relationship between leader PSRB and follower PSRB.

Hypothesis 4: There will be a three-way interaction between leader PSRB, empowering leadership and courage, such that the positive relationship between leader PSRB and follower PSRB is the strongest when empowering leadership and courage are simultaneously high.

## Materials and Methods

### Participants

Data were collected from part-time graduate students at a university in Guangzhou, China. They were all full-time employees, and each had one immediate supervisor. To avoid the potential for same source common-method bias, we collected data from two sources: students and their immediate supervisors.

The students were invited to participate during their organizational behavior class. Before distributing the questionnaires, the potential participants were informed of the purpose of the survey, and it was stressed that participation was voluntary. We promised that their responses would be confidential and that their data would be used only for research purposes. After obtaining consent, we distributed the printed questionnaires to the participants and asked them to report their demographic information, perceptions of the extents of empowering leadership, courage and PSRB. After collecting the completed questionnaires, we gave those students an envelope marked with “For Your Immediate Supervisor,” and asked them to take back the envelope to their supervisors to complete. Such envelope contained a questionnaire measuring supervisors’ PSRB, and a cover letter explaining to supervisors that participation in this research was voluntary and the data they provided would be confidential. We asked students to bring back the envelope completed by their supervisors next class (a week after).

Altogether, 224 leader-follower dyad questionnaires initially were distributed. After dropping the incomplete questionnaires, 174 leader-follower pairs remained (174 leaders and 202 followers with response rates of 77.7 and 90.2%, respectively). Of the followers, 63.8% were male, the mean age was 29.10 years (*SD* = 3.90), and the average tenure was 6.05 years (*SD* = 3.98). The followers had been working with their leaders for an average of 2.99 years (*SD* = 2.17). About 42.5% of the leader sample was male with an average age of 40.53 years (*SD* = 6.29).

### Measures

All English-language items in the questionnaire were translated into Chinese using the translation/back-translation procedure, and a Likert-type scale was used for the response options on all the items, where 1 = *strongly disagree* to 5 = *strongly agree*.

PSRB: PSRB (leader and follower) was measured using the General Pro-Social Rule Breaking Scale (GPSRBS) developed by Dahling et al. (2012). The scale comprised 13 items in three sub-scales: (a) the efficiency subscale (five items, e.g., “I break organizational rules or policies to do my job more efficiently”), (b) the coworker assistance subscale (four items, e.g., “When another employee needs my help, I disobey organizational policies to help him/her”) and (c) the customer assistance subscale (four items, e.g., “I break organizational rules to provide better customer service”). However, some of the participants in our study did not work in the service sector, so we omitted the third sub-scale. Cronbach’s alpha for this scale was 0.93 among the 174 leaders and 0.87 among the 202 followers.

Empowering leadership: Empowering leadership was measured using Ahearne et al.’s (2005) ten-item scale. This scale contains four multi-item subscales that focus on: (a) enhancing the meaningfulness of work (e.g., “My supervisor helps me understand how my objectives and goals relate to that of the company”), (b) fostering participation in decision making (e.g., “My supervisor makes many decisions together with me”), (c) expressing confidence in high performance (e.g., “My supervisor believes that I can handle demanding tasks”), and (d) providing autonomy from bureaucratic constraints (e.g., “My supervisor allows me to do my job my way”). Cronbach’s alpha of the scale was 0.87.

Courage: Courage was measured using the eight-item scale developed by Woodard and Pury (2007). Sample items include “I would risk rejection by important others for a chance at achieving my life goals”. Cronbach’s alpha of the scale was 0.73.

Control variables: Previous studies on PSRB included follower gender, age, and organizational tenure as control variables (Huang et al., 2014). Consequently, we controlled for the effects of these variables in the analysis. In addition, we controlled for the effect of dyadic tenure, which was defined as “the period in which a follower had worked with his or her leader.” Age, organizational tenure, and dyadic tenure were measured in years. Gender was a dummy variable coded “0 = *female*” and 1 = *male*.”

### Analyses

First, descriptive statistics were generated (means, standard deviations, and bivariate correlations). Next, Multiple Moderated Regressions (MMR) was performed to test the hypotheses. Specifically, the control variables (gender, age, organizational tenure and dyadic tenure) were entered into the equation in Step 1, to which the key independent variable (leader PSRB) and two moderators (empowering leadership and courage) were added in Step 2. In Step 3, we entered three interaction terms: leader PSRB × empowering leadership, leader PSRB × courage, and empowering leadership × courage. Finally, a three-way interaction term (leader PSRB × empowering leadership × courage) was included in Step 4. To avoid problems of multicollinearity, all of the independent variables were mean-centered before the interaction terms were constructed (Aiken and West, 1991).

## Results

### Descriptive Statistics and Correlations

Table 1 shows the means, standard deviations, correlation coefficients (*r*), and reliability statistics of the variables. Gender, age, organizational tenure, and dyadic tenure were not significantly correlated with follower PSRB. Of interest, leader PSRB was positively correlated with follower PSRB (*r* = 0.22, *p* < 0.01), which provided preliminary support of Hypothesis 1.

**Table 1:** Means, standard deviations, and correlations among study variables.

Variable	Mean	*SD*	1	2	3	4	5	6	7	8
1. Gender	0.64	0.48	–							
2. Age	29.10	3.90	–0.06	–						
3. Organizational tenure	6.05	3.98	–0.03	0.87***	–					
4. Dyadic tenure	2.99	2.17	0.01	0.34***	0.21**	–				
5. Leader PSRB	2.15	0.68	–0.01	–0.06	0.03	–0.01	(0.93)			
6. Empowering leadership	3.40	0.66	0.11	0.16*	0.21**	0.07	0.19*	(0.87)		
7. Courage	3.09	0.53	0.01	0.14	0.14	0.12	0.07	0.24**	(0.73)	
8. Follower PSRB	2.13	0.63	0.01	0.08	0.04	0.05	0.22**	0.15	0.20**	(0.87)


*N = 174. SD, standard deviations. Reliability coefficients are reported along the diagonal.^∗^p < 0.05, ^∗∗^p < 0.01, ^∗∗∗^p < 0.001.*

### Hypothesis Testing

Hypotheses 1 predicted that leader PSRB positively related to follower PSRB. The results of Model 2 indicated that, as predicted, leader PSRB positively related to follower PSRB (β = 0.20, *p* < 0.01, Model 2). Thus, Hypotheses 1 was supported.

**FIGURE 2:**
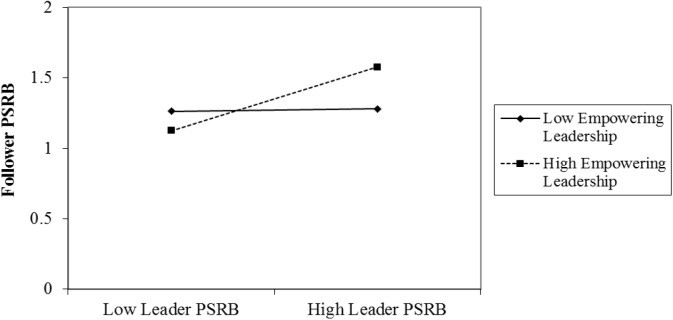
Interaction effects of leader PSRB and empowering leadership on follower PSRB.

Hypothesis 2 predicted that empowering leadership moderated the positive relationship between leader PSRB and follower PSRB, such that it would be stronger when empowering leadership was high. Model 3 in Table 2 showed that the interaction effect of leader PSRB and empowering leadership on follower PSRB was significant (β = 0.24, *p* < 0.05). Figure 2 shows that the association between leader PSRB and follower PSRB was contingent upon the level of empowering leadership. We also performed a simple slope test, which found that, when empowering leadership was high, leader PSRB was positively related to follower PSRB (simple slope = 0.33, *p* < 0.001). However, no significant relationship between leader PSRB and follower PSRB was found (simple slope = 0.01, ns) when empowering leadership was low. Hence, Hypothesis 2 was supported.

**Table 2:** Results of moderated regression analyses.

	Follower PSRB			
Predictor variable	Model 1	Model 2	Model 3	Model 4
Intercept	1.32	1.06	1.31	1.43
**Control variables**				
Gender	0.02	0.01	–0.06	–0.05
Age	0.03	0.05	0.04	0.03
Organizational tenure	–0.02	–0.04	–0.03	–0.03
Dyadic tenure	0.00	0.00	–0.02	–0.02
**Independent variable**				
Leader PSRB		0.20^∗∗^	0.17^∗^	0.13
**Moderators**				
Empowering leadership		0.08	0.06	0.03
Courage		0.19^∗^	0.17	0.14
**Interaction terms**				
Leader PSRB × Empowering leadership			0.24^∗^	0.23^∗^
Leader PSRB × Courage			0.31^∗^	0.35^∗^
Empowering leadership × Courage			–0.04	–0.13
Leader PSRB × Empowering leadership × Courage				0.41^∗^
*R*^2^	0.01	0.10	0.17	0.20
Δ*R*^2^	–	0.09	0.07	0.03
*F*	0.51	2.70^∗^	3.40^∗∗∗^	3.70^∗∗∗^


*N = 174. Unstandardized regression coefficients are reported. ^∗^p < 0.05, ^∗∗^p < 0.01, ^∗∗∗^p < 0.001.*

**FIGURE 3:**
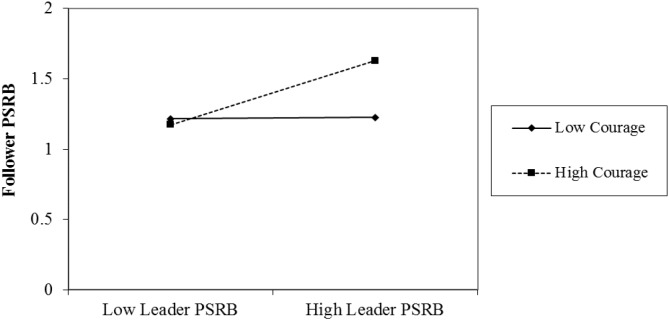
Interaction effects of leader PSRB and courage on follower PSRB.

Hypothesis 3 stated that courage moderated the positive relationship between leader PSRB and follower PSRB, such that the relationship would be stronger when courage was high. The results indicated that the interaction between leader PSRB and courage significantly related to follower PSRB (β = 0.31, *p* < 0.05, Model 3). The subsequent simple slope analysis and Figure 3 found a positive moderating effect of courage on the relationship between leader PSRB and follower PSRB. As shown in Figure 3, leader PSRB had a positive relationship with follower PSRB when courage was high (simple slope = 0.34, *p* < 0.001). However, leader PSRB was not significantly related to follower PSRB when courage was low (simple slope = 0.01, ns). Therefore, Hypothesis 3 was supported.

**FIGURE 4:**
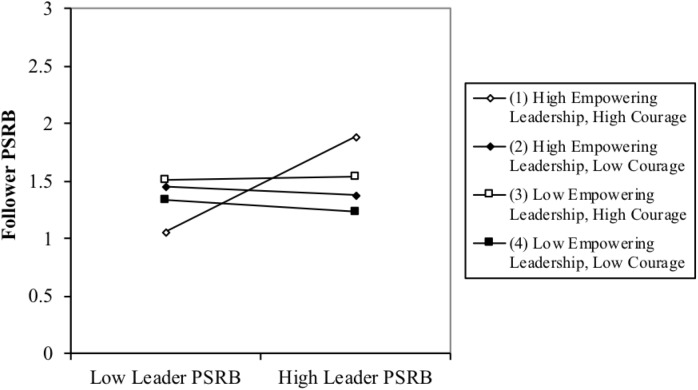
Interaction effects of leader PSRB, empowering leadership and courage on follower PSRB.

Hypothesis 4 predicted a three-way interaction among leader PSRB, empowering leadership, and courage, such that the positive relationship of leader PSRB and follower PSRB would be strongest when empowering leadership and courage were high. Model 4 in Table 2 showed that the leader PSRB × empowering leadership × courage three-way interaction term was significant for follower PSRB (β = 0.41, *p* < 0.05, Model 4). This significant interaction was illustrated in Figure 4. As hypothesized, the positive relationship between leader PSRB and follower PSRB was strongest for high levels of empowering leadership and courage (simple slope = 0.60, *p* < 0.001). In contrast, the slope was not significant under the three other conditions (see Table 3). Therefore, Hypothesis 4 was supported.

**Table 3:** Results of moderated regression analyses.

Interaction	Moderator condition	β (*p*-value)
Leader PSRB × EL	High EL	0.33 (*p* < 0.001)
	Low EL	0.01 (n.s.)
Leader PSRB × Courage	High courage	0.34 (*p* < 0.001)
	Low courage	0.01 (n.s.)
Leader PSRB × EL × Courage	High EL, high courage	0.60 (*p* < 0.001)
	High EL, low courage	-0.05 (n.s.)
	Low EL, high courage	0.02 (n.s.)
	Low EL, low courage	-0.07 (n.s.)


*N = 174. EL, Empowering leadership.*

## Discussion

Drawing on social learning theory and humanistic cognitive behaviorism theory, we conducted an empirical study on the effects of leader PSRB on follower PSRB. We also examined whether this effect was moderated by the extents of empowering leadership and courage. The results of the MMR revealed that: (1) leader PSRB positively related to follower PSRB; (2) empowering leadership and courage had independent positive moderating effects on this relationship, such that when empowering leadership or courage was high, leader PSRB had a relatively stronger influence on follower PSRB. (3) the leader PSRB-follower PSRB relationship was jointly moderated by empowering leadership and courage, such that the positive relationship between leader PSRB and follower PSRB was strongest when both empowering leadership and courage were high.

### Theoretical Implications

First, this empirical study found a trickle-down effect of leader PSRB on follower PSRB. Because of their relatively high hierarchical positions and strong authority in organizations, leaders might be extraordinarily attractive to followers. Therefore, their behaviors and the values behind them might easily become models that followers learn and imitate. When followers observe leader PSRB, they tend to believe that those behaviors are tolerated by the organization, and they are likely to imitate them when dealing with similar situations. Our study’s results not only support the view that “leaders are an important factor influencing follower behaviors” (Bavik et al., 2018), they also provide empirical evidence in support of social learning theory. Furthermore, we supplemented the literature on trickle-down effects of leader behaviors (e.g., Yaffe and Kark, 2011; Liu et al., 2012; Liden et al., 2014).

Second, this study introduced empowering leadership and found a moderating influence in the relationship between leader PSRB and follower PSRB. Previous research has found that the extent of empowering leadership helped followers to gain confidence in their abilities and that it provided an unrestricted and autonomous environment (Spreitzer and Doneson, 2005). With high levels of self-efficacy and job autonomy, followers are likely to learn that they can deviate from organizational rules in some instances, and, therefore, the likelihood that they will perform PSRB might increase. The result provides new evidence for the claim that “empowering leadership can promote followers to perform discretionary behaviors” (Zhong et al., 2011).

Third, our study’s results supported the moderating role of courage. Courage, as a stable individual characteristic, plays an important part in developing PSRB. Followers high in courage were relatively more positive and confident regarding the possible consequences of performing PSRB, which is why, when the option of breaking the rules arose, they were more likely to perform PSRB. Previous studies have found that personality influenced individuals’ willingness to break the rules (Judge et al., 2006), and personal traits, such as empathy, boldness (Morrison, 2006), and conscientiousness (Dahling et al., 2012), influenced follower PSRB. Our results not only supported those previous findings, they also contributed to the literature on the relationship between personality and PSRB.

Finally, based on Humanistic Cognitive Behavioral Theory, we hypothesized and confirmed a three-way interaction effect on follower PSRB. When follower courage and empowering leadership were simultaneously high, followers were most likely to learn from leaders and perform PSRB because they had firm beliefs, high self-efficacy, and a relaxing and unrestricted work environment that encouraged PSRB. However, when follower courage and empowering leadership were simultaneously or individually low, followers were less likely to engage in PSRB because they had low expectations for the positive results of their actions and/or they worked in oppressive environments. The results of this study not only provide new support for Humanistic Cognitive Behavioral Theory; they also corroborate the perspective that individual differences and organizational factors influence the behaviors of organizational members (Robertson and Callinan, 1998).

### Practical Implications

Our findings have several important practical implications. First, when rule breaking is considered beneficial to their organizations, the organizations can capitalize on the influence that leader PSRB has on followers to encourage followers to break out of any shackles and find out new ways and approaches. However, organizations should also limit the frequency and scope of leader PSRB to indirectly control the extent of follower PSRB.

Second, given that followers are most likely to imitate and learn leader PSRB when the extents of follower courage and empowering leadership are high, organizations should focus on hiring and promoting candidates who are more courageous as those individuals have firm beliefs, can effectively control their fears, and remain optimistic in the face of uncertainty. Moreover, managers should enhance autonomy, unrestricted workplace environments, and encourage employee confidence to increase the likelihood that followers will violate the rules when doing so seems to benefit the organization.

### Limitations and Future Directions

This study has a few limitations. First, the design was cross-sectional, which limited our ability to draw causal conclusions. Therefore, longitudinal designs or situational experiments are suggested for extensions of this study. Second, the sample was limited to Guangdong Province, and it is not clear whether our findings can be generalized to other areas of China or other cultures. To improve the external validity, future studies should employ representative samples of larger populations. Third, we only tested the boundary conditions in which leaders’ PSRB promotes followers’ PSRB. According to social learning theory, as leaders perform PSRB, followers will learn the value criteria embodied in PSRB, i.e., the organizational rules can be broken to facilitate the welfare of the organization and its stakeholders. Such value criteria could further trigger pro-organizational motivation of followers. Hence, future studies can test the mediating role of pro-organizational motivation.

## Ethics Statement

The study was approved by the ethical committee of Guangzhou University. We obtained informed oral consent from students before data collection, and the consent obtained from their supervisors was both written and informed. Both students and their supervisors were given the opportunity to refuse to participate, to omit questions or to withdraw from the study at any time without penalization.

## Author Contributions

YC contributed to developing the theoretical framework, data analysis, and overall writing of the paper. LW, XL, and HC contributed to the revision of the paper as well as the overall design. YH and HY contributed to the data collection.

## Conflict of Interest Statement

The authors declare that the research was conducted in the absence of any commercial or financial relationships that could be construed as a potential conflict of interest.
